# Vulvar Lymphangioma Arising in the Setting of May–Thurner Syndrome

**DOI:** 10.1155/2024/9761009

**Published:** 2024-09-18

**Authors:** Surekha Bantumilli, Ian Flyke, Muthu Kumar Sakthivel, Christine E. Bookhout

**Affiliations:** ^1^ Department of Pathology and Laboratory Medicine University of North Carolina at Chapel Hill, Chapel Hill, North Carolina, USA; ^2^ Department of Obstetrics and Gynecology University of North Carolina at Chapel Hill, Chapel Hill, North Carolina, USA; ^3^ Department of Radiology University of North Carolina at Chapel Hill, Chapel Hill, North Carolina, USA

**Keywords:** lymphangioma, May–Thurner syndrome, thrombosis, vulva

## Abstract

This case report describes an instance of vulvar lymphangioma occurring in the setting of May–Thurner syndrome (MTS), an association between two vascular conditions that we do not believe has been previously reported. Lymphangioma, also known as lymphatic malformation, is a benign lesion typified by dilatation of endothelial-lined lymphatic channels involving the skin and subcutis, which can occur either as a congenital abnormality or as a result of acquired damage to lymphatic channels. Lymphangioma is a rare lesion in the vulva. MTS, also known as iliac vein compression syndrome or Cockett's syndrome, is a condition of left iliac vein obstruction due to overriding the right common iliac artery which can lead to iliofemoral deep vein thrombosis. In this report, we describe the case of a 29-year-old woman with MTS diagnosed at 7 years of age with poor lymphatic drainage and pelvic pain requiring left iliac vein stenting. She presented with left vulvar discomfort and chronic lower extremity edema and was found to have warty vulvar masses, with histopathological examination showing lymphangioma of the vulva. We believe that this is the first report of vulvar lymphangioma recognized in the setting of MTS, and we will discuss the clinical features, etiology, and possible pathophysiologic association between these two entities.

## 1. Introduction

Lymphangioma circumscriptum of the vulva is a rare and benign lymphatic lesion that is frequently underrecognized and often occurs in patients with a history of pelvic radiotherapy or malignancy. Also known as microcystic lymphatic malformation, it is composed of a fluid-filled cystic proliferation of benign malformed lymphatic channels that may not communicate with the normal lymphatic system [1]. The etiology of lymphangioma is not entirely understood; it can be either congenital or acquired due to injury to lymphatic vessels or disruption of lymphatic drainage. Potential causes of lymphatic system compromise or lymphedema include lymph node dissection, radiation induced injury, inflammation, fibrosis, and malignant or mechanical obstruction [2]. The most common sites are the tongue, mouth, chest, and axilla with retroperitoneum also reported [1, 3]; the vulva is an infrequently involved site. We report a rare presentation of acquired vulvar lymphangioma associated with chronic lymphedema and pelvic congestion syndrome in the setting of May–Thurner syndrome (MTS) in a 29-year-old female. The presence of classic symptoms of lymphatic obstruction in conjunction with MTS raises the possibility that MTS could have been the triggering event for the vulvar lymphangioma in this case.

## 2. Case Report

A 29-year-old female patient presented to our institution with complaints of chronic lower extremity edema and vulvar discomfort. The patient's medical history is noteworthy for MTS since her childhood for which she was on antithrombotic therapy (enoxaparin during pregnancy and aspirin thereafter). Her surgical history is significant for vascular surgery with iliac vein stent in place for 23 years with one revision 16 years later as well as atrial septal defect closure. Despite her iliac vein stent, the patient continued to complain of increasing lymphedema and consulted vascular surgery, who recommended starting baby aspirin and comprehensive decompressive therapy for lymphedema. The patient noticed a labial rash but thought it might be an allergic reaction. During a recent cesarean section delivery, the patient's obstetrician noted bilateral labial verrucous papular lesions and recommended dermatologic follow-up. Clinically, the initial differential diagnosis entertained for this lesion included possible genital warts or squamous cell carcinoma. The lesion was biopsied at a dermatology clinic and diagnosed as lymphangioma with ulceration and acute inflammation. Subsequently, she transferred her care to our institution's gynecological service for further management.

**Figure 1:**
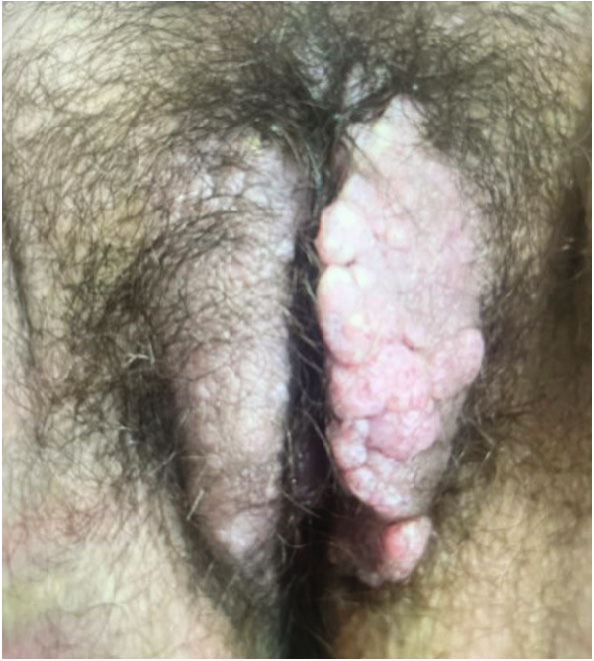
Clinical presentation of vulvar lymphangioma: wart-like projections of the labia majora and minora predominantly involving the left more than right.

On genital examination, there was significant induration and wart-like projections of the labia majora and minora extending the length of the labia as well as 3–4 cm superficially into the mons bilaterally, sparing the clitoris (Figure 1). On speculum and bimanual examination, the cervix and uterus were unremarkable. Her musculoskeletal examination was significant for nonpitting edema of the bilateral lower extremities.

**Figure 2:**
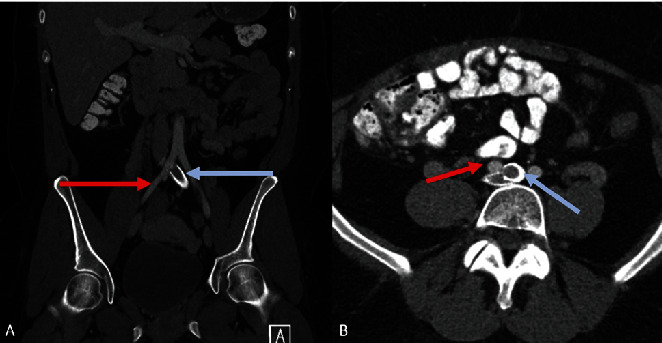
May–Thurner syndrome with (A) coronal contrast-enhanced CT abdomen demonstrating that the right common iliac artery (red arrow) compresses the proximal left common iliac vein containing the venous stent (blue arrow) in a different plane. (B) Axial contrast-enhanced CT abdomen illustrates that the right common iliac artery (red arrow) compresses the proximal left common iliac vein containing the venous stent (blue arrow) against the L4 vertebral body.

**Figure 3:**
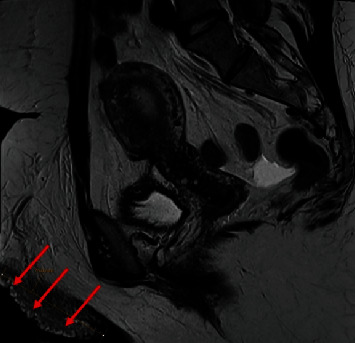
MRI pelvis sagittal T2 weighted image demonstrates 7.5 cm completely extra-pelvic hyperintense lobulated multicystic lesion (red arrow) in the left labia majora (vulvar lymphangioma).

Contrast-enhanced CT scan of the abdomen demonstrated mass effect caused by the right common iliac artery overlying the left common iliac vein with a vascular stent. This mass effect resulted in decreased anteroposterior luminal diameter of the left common iliac vein and compression of the vein against the anterior margin of the L4 vertebral body (Figure 2). MRI of the pelvis with and without contrast demonstrated a T2 hyperintense extra-pelvic subcutaneous lobulated multicystic lesion measuring 7.5 cm in the left vulva. On the T2 fat suppressed images, there was evidence of edema and a multicystic lesion in the left greater than the right vulva. On post contrast images, the 7.5 cm left vulvar lesion appeared hypointense with no demonstrable contrast enhancement (Figure 3). Susceptibility artifact was present related to the left common iliac vein stent. The bilateral internal iliac, external iliac, common femoral, femoral, and deep femoral veins were patent. Prominent uterine arteries were also seen measuring up to 15 mm on the right and 7 mm on the left with moderately enlarged reactive bilateral inguinal lymph nodes and no demonstrable evidence of compression of lymph nodes.

**Figure 4:**
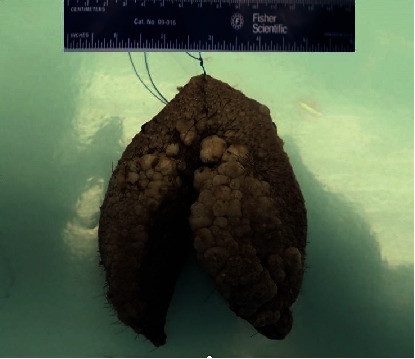
Gross resection specimen of partial vulvectomy showing verrucous lesion.

Following her assessment, management options were discussed, and she was informed of the possibility of recurrence of lymphangioma after resection. The patient did elect to have surgical resection, and bilateral partial vulvectomy was performed. Grossly, the left and right labia majora displayed an ill-defined gray-white warty mass measuring 7.9 × 4.5 × 0.9 cm involving the left side more than the right (Figure 4). The cut surface of the lesion appeared flesh colored with white discoloration superficially, and it appeared confined to the skin without underlying invasion.

**Figure 5:**
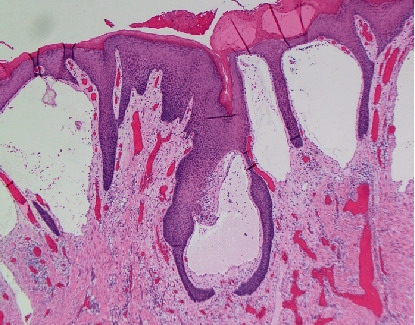
Higher power magnification of the lesion showing dilated lymphatic spaces containing eosinophilic proteinaceous material and lymphocytes. Note the accompanying hyperkeratotic changes. (H&E 100x.)

**Figure 6:**
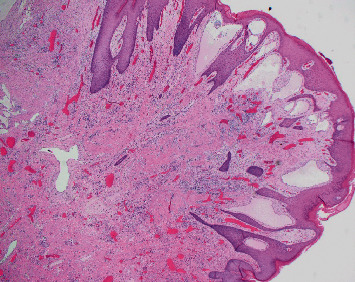
Lower power magnification with dilated lymphatic channels, variably sized. (H&E 20x.)

Histopathological examination of the vulvectomy specimen confirmed variably sized dilated lymphatic spaces with associated mixed inflammation, consistent with a diagnosis of lymphangioma. There were multifocal areas of fibrosis and collections of histiocytes (Figure 5). The deep and peripheral resection margins were uninvolved. The dilated lymphatic spaces contained intraluminal eosinophilic proteinaceous material and lymphocytes. Additionally, the skin showed hyperkeratosis with epithelial and stromal reactive changes (Figure 6). No evidence of any dysplasia or carcinoma was identified.

Postoperatively, the patient healed well and was referred to skilled lymphedema therapy with recommendation to continue compression garments for her complaints of lymphedema. Unfortunately, she has continued to be seen in the emergency room for symptoms of severe pelvic congestion syndrome. Nevertheless, as of the time of this report, there has been no recurrence of her lymphangioma after resection.

## 3. Discussion

To our knowledge, this is the first described case of vulvar lymphangioma/lymphatic malformation occurring in the setting of MTS. Lymphangioma circumscriptum is a benign lesion of malformed lymphatic vessels that was first described by Fox and Fox in 1870 as “lymphangiectodes” and later termed “lymphangioma circumscriptum” in 1889 by Malcolm Morris. Clinically, the lymphangioma has been described as having an appearance of “frogspawn” with vesicular lesions that drain clear fluid [2]. The abnormal lymphatic vessels may not communicate with the remainder of the body's lymphatic system and become dilated and filled with fluid [1, 4]. Anatomically, the cutaneous and subcutaneous tissues of the vulva drain primarily to superficial inguinal nodes, which drain into deep inguinal lymph nodes [5, 6]. Deep inguinal nodes drain into the external iliac lymph nodes, which then drain into the common iliac lymph nodes and para-aortic lymph nodes [6]. Since the common iliac nodes are located in close proximity to the common iliac vessels just distal to the bifurcation of the aorta in front of the fourth or fifth lumbar vertebra, compression of this region in MTS could conceivably inhibit lymphatic drainage from the vulva.

In 1970, this vascular entity was classified clinically into two categories as classic type or localized type based on its presentation. Classic type is identified around or after birth with a predilection to proximal locations, whereas localized type can occur at any age with no site predilection. Congenital lymphangiomas have been linked with chromosomal disorders like Down, Noonan, Turner, Edwards, and Patau syndromes. A thorough physical examination in the appropriate clinical setting as well as a high index of suspicion, with biopsy performed as appropriate, is key in establishing the diagnosis [4].

Acquired lymphangioma is related to obstruction of lymphatic vessels and is most commonly seen in association with prior malignancies of the anogenital and pelvic region [2], with radiation or surgery likely causing lymphatic damage. In the study by Luu et al. of malignancy-associated acquired vulvar lymphangiomas, 70% of cases had a history of lymph node dissection, although 30% of cases did not; 91.4% had undergone radiation treatment [2]. Other less common proposed causes include infection and inflammation [3, 7], and occasional cases have been reported in association with inflammatory bowel disease [7]. The role of specific vascular endothelial growth factors and receptors in the occurrence of vulvar lymphangioma has not been fully determined, although interruption in the integrity of lymphatic drainage seems to play an instrumental role.

MTS is an anatomic anomaly leading to chronic compression of the left common iliac vein by the right common iliac artery against a lumbar vertebral body (commonly involving the fifth vertebra). The spectrum of symptoms is variable, ranging from asymptomatic throughout the patient's lifetime to symptomatic with lower extremity edema, thrombotic syndromes, and lymphedema. Females are more susceptible of this vascular condition due to their anatomy [8]. In 1957, May and Thurner performed a comprehensive study on 430 cadavers that were proven to have left-sided lower limb deep vein thrombosis and recognized that 22% of cadavers demonstrated a “spur-like” projection in the venous lumen at the compression site [8] which led to the discovery of this syndrome.

MTS is common in young females and is considered to be responsible for approximately 2–3% of all deep vein thrombosis [9]. MTS is believed to be significantly underdiagnosed, given that many patients with this syndrome are asymptomatic and only develop deep vein thrombosis when exposed to additional inciting factors like oral contraceptive use, immobility, and inherent thrombophilia [10]. The majority of the patients are unrecognized until serious complications occur. Detecting these vascular variants early is imperative, since a delay in diagnosis increases the risk of DVTs and may lead to devastating complications like pulmonary embolism [11].

The most common presenting symptoms are left lower extremity deep vein thrombosis and lymphedema. Venous obstruction should also be considered in patients presenting with nonthrombotic symptoms like lower extremity or pelvic discomfort, pelvic varicosities, changes in skin, and stasis ulcers [9, 12]. Diagnosis is established based on clinical manifestations and imaging studies. Specifically, conventional venography is widely employed for diagnosis, although CT or MRI venography or intravascular ultrasound can also be used [8]. Endovascular and thrombolytic therapies are currently the mainstay of treatment to prevent complications of MTS [10]. Conservative measures like exercise, physical therapy, and compression garments can be attempted to treat lymphedema. If initial measures fail, surgical options such as lymphedema surgery, suction-assisted lipectomy, lymphatic-venous anastomosis, or vascularized lymph node transfer surgery may be recommended [12].

Although therapy can help prevent complications, in our patient's case, there was significant narrowing of the left iliac vein by the right iliac artery despite stenting, and she has had ongoing issues with persistent lymphedema and constant pelvic pain. Interestingly, the proposed mechanistic basis for the lymphatic obstruction in MTS is that venous endothelial damage precipitates obstruction and stasis due to constant iliac arterial pulsation at the compression site. This repetitive cycle of injury and repair may trigger collagen and elastin accumulation which consequently blocks venous drainage [8, 13] and can advance to a spur-like formation in the lumen of the vein, causing subsequent permanent damage [10].

With regards to the second vascular lesion in our case, vulvar lymphangiomas are rarely reported and likely are underdiagnosed due to lack of recognition. Some factors that may confound successful diagnosis include clinical presentation that mimics other entities, delayed development of lymphangioma years after treatment of cancer, and lack of awareness regarding this entity [2]. Clinically, the lesion can show fluid-filled papules or vesicles or can appear warty secondary to hyperkeratosis; the clinical symptoms range from asymptomatic to discharging fluid, itching, and pain. The extent of the lesion can vary from small foci to extensive bilateral vulvar involvement, sometimes spreading to the thigh or mons pubis [1, 4]. The differential diagnosis may include genital warts (condyloma acuminatum), herpes infection, molluscum contagiosum, vulvar dysplasia or carcinoma, and leiomyoma [2, 14]. When biopsied or resected, microscopic diagnosis is generally straightforward. Characteristic features include dilated lymphatic channels in the epidermal and superficial dermal region lined by flat endothelial cells and containing proteinaceous fluid, sometimes with red and white blood cells [14]. The immunohistochemical stain D2-40 highlights these lymphatic channels and aids in diagnosis [2].

Vulvar lymphangioma is associated with significant symptoms such as vulvar edema and pain as well as cosmetic concerns, psychosexual dysfunction, and complications such as cellulitis [10]. The main goal of treatment is to mitigate symptoms and avoid recurrence, which can be achieved by complete removal of abnormal lymphatics. However, there are no standard guidelines for therapy. Surgical excision, varying from biopsy to radical vulvectomy, is a cornerstone of treatment. Unfortunately, recurrence after surgery is common [15]. Further options for treatment include ablative laser treatment or destructive methods like sclerotherapy, electrocoagulation, radiofrequency current coagulation, and cryotherapy [2, 16]. Malignant transformation of lymphangioma is rare but has been reported [17].

Based on our literature review and the evidence of lymphatic obstruction in our patient, we believe that the vascular anomaly of MTS was likely an inciting factor for the development of her vulvar lymphangioma and favor a causative association in this case [4, 12].

## 4. Conclusion

Although vulvar lymphangiomas have rarely been previously reported in the literature, our case report is unique in that we describe a case where this benign vascular lesion is present in association with MTS, and we propose a causal link between these two entities. Given this association, we encourage our clinical colleagues to have a high index of suspicion and to consider MTS in the differential diagnosis of patients with vulvar lymphangioma or evidence of vulvar lymphatic obstruction. We also recommend keeping vulvar lymphangioma in mind as a diagnostic consideration in cases with vesicular or warty vulvar lesions, especially in patients with a history of cancer or vascular or lymphatic compromise.

## Data Availability

The authors have nothing to report.
